# Svalbard winter warming is reaching melting point

**DOI:** 10.1038/s41467-025-60926-8

**Published:** 2025-07-21

**Authors:** James A. Bradley, Laura Molares Moncayo, Gabriella Gallo, Jacopo Brusca, Tessa Viglezio, Jacopo Pasotti, Donato Giovannelli

**Affiliations:** 1 Aix Marseille Université, Université de Toulon, CNRS, IRD, MIO, Marseille, France; 2 School of Biological and Behavioural Sciences, Queen Mary University of London, London, UK; 3 School of Geography, Queen Mary University of London, London, UK; 4 Natural History Museum, London, UK; 5 Dipartimento di Biologia, Università degli Studi di Napoli Federico II, Naples, Italy; 6 National PhD program in Polar Sciences, Ca’ Foscari University of Venice, Venice, Mestre Italy; 7 Istituto di Scienze Polari, Consiglio Nazionale delle Ricerche, Venice, Mestre Italy; 8 Istituto per le Risorse Biologiche e le Biotecnologie Marine, Consiglio Nazionale delle Ricerche, Ancona, Italy; 9 Department of Marine and Coastal Science, Rutgers University, New Brunswick, New Jersey USA; 10 Marine Chemistry & Geochemistry, Woods Hole Oceanographic Institution, Falmouth, Massachusetts, USA; 11 Earth-Life Science Institute (ELSI), Tokyo Institute of Technology, Tokyo, Japan

**Keywords:** Cryospheric science, Biogeochemistry

## Abstract

The Arctic winters are changing fast. In February 2025, Svalbard endured rain, thawing tundra, and pooling meltwater. The Comment by Bradley and coauthors describes how winter warming is reshaping polar ecosystems—and why this resembles the new Arctic.

## Teaser

The Arctic is at the forefront of global climate change and is encountering unprecedented winter warming. In February 2025, exceptionally high air temperatures and rainfall over Svalbard triggered widespread snowmelt and pooling of meltwater. Increasingly frequent winter thaw events are reshaping Arctic landscapes, signaling a dramatic shift towards a new Arctic.

## Accelerated winter warming and wetting

Svalbard is at the front line of the climate crisis, warming at six to seven times the global average rate^1^. Human-caused global warming is particularly amplified in the Arctic, causing the climate in the Arctic to warm more quickly than the rest of the Earth^2^. The winter period is experiencing the highest rates of warming^3^ with winter temperatures over Svalbard rising at nearly twice the annual average^4–6^. Meanwhile, centennial trends for annual precipitation in west Svalbard show increases of 3–4 % per decade, of which a greater proportion is falling as rain^7^. As such, over the past 40 years, rain-on-snow events have significantly increased, and rain is projected to become the dominant form of precipitation in the Arctic by the end of this century^7^.

**Fig. 1:**
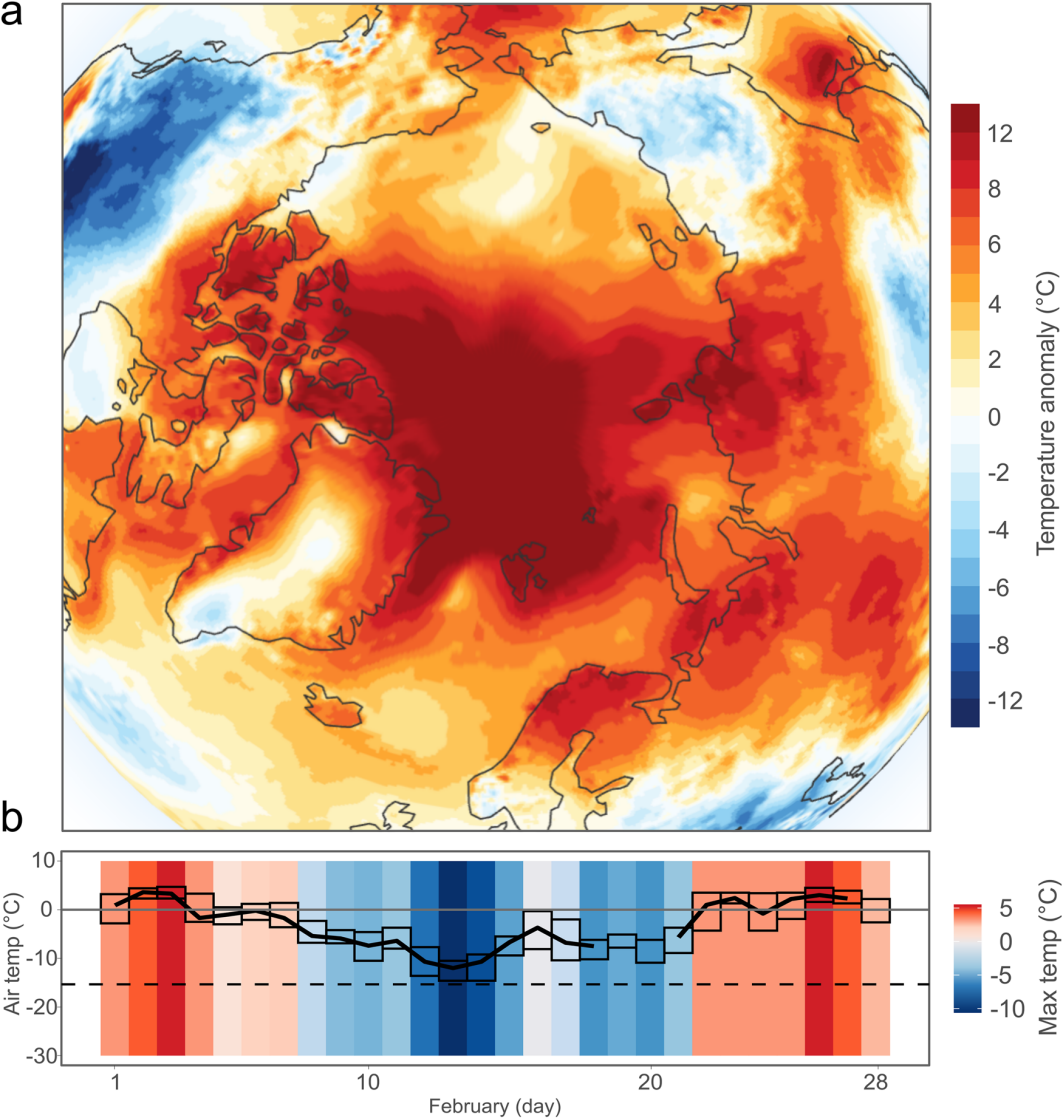
Warming over Svalbard during February 2025. **a** Surface air temperature anomaly for February 2025 over the Arctic region relative to the February average for the period 1991–2020. Data source: ERA5^8^, obtained from the Copernicus Climate Change Service (C3S), implemented by the European Center for Medium-Range Weather Forecasts (ECMWF)^24^. **b** Daily air temperatures for February 2025 from Ny-Ålesund observation station, Svalbard, elevation 8 m, established in July 1974. The solid black line is the daily average. The black boxes represent the minimum and maximum temperatures recorded. The horizontal dashed black line represents the average air temperatures for Longyearbyen, Svalbard, for the month of February between 1959–2001, estimated from the instrumental Svalbard Airport series^9^. Background stripes are colored according to the daily maximum temperature.

This year, Arctic winter air temperatures were among the warmest ever recorded^8^. In Ny-Ålesund, the world’s northernmost permanent settlement, situated in north-west Svalbard and approximately 1,200 km from the North Pole, the air temperature average for February 2025 was -3.3 °C — considerably higher than the 1961-2001 average for this time of year of -15 °C (Fig. 1a)^9^, and reached a maximum of 4.7 °C. Air temperatures higher than 0 °C were recorded in Ny-Ålesund on 14 of the 28 days of February 2025 (Fig. 1b). Such sustained warmth, coupled with prolonged rainfall, triggered widespread melting of snow and ice. When winter warming crosses the 0 °C threshold, it marks more than just a warm anomaly — it signals a fundamental shift in Arctic winter dynamics. Episodic thawing events during winter can have significant and lasting environmental consequences, including influencing ice layer formation, triggering microbial activation, altering nutrient discharge, and affecting permafrost thaw and ground ice development. The episodic warming event of February 2025 was not an isolated occurrence: winter warming events in Svalbard have been a recurring phenomenon in recent decades as a consequence of anthropogenic climate change^3^. As climate change continues to take hold, the frequency and magnitude of winter warming episodes will increase, and so will their effects on Arctic systems and societies.

Our teams are working in Ny-Ålesund to study glacial and terrestrial microbial communities and their role in carbon and other elemental cycles during the dark, frozen, winter period, for which data remains scarce^10^. Our winter-time field campaigns in Svalbard are conducted under the expectation of sub-zero temperatures and extensive snow cover — conditions that have historically been typical in Svalbard during winter, but in recent years are increasingly threatened by climate change. However, in February 2025, we encountered air temperatures persistently above 0 °C, as well as rainfall, exceptionally low snow cover, and pooling meltwater covering the tundra. Wintertime warming and rain turned Ny-Ålesund and the surrounding landscape into a melting ice rink, disrupting planned sampling efforts, forcing adaptations in methodologies, provoking new scientific questions, and raising concerns about the long-term feasibility of our winter research practices and logistics under increasingly variable conditions and rising temperatures.

## The new Arctic

**Fig. 2:**
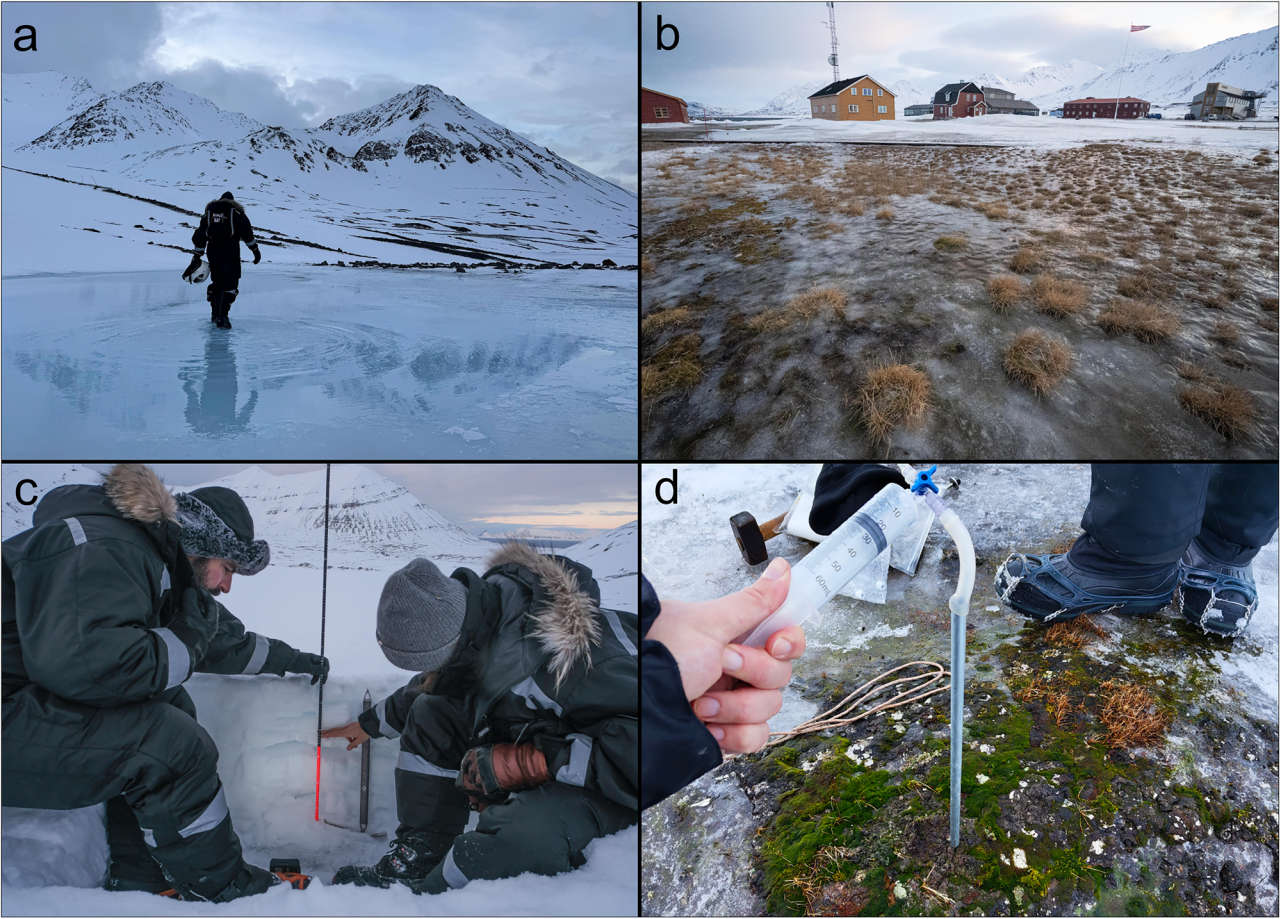
Snapshots from the February 2025 thawing event in Ny-Ålesund, Svalbard. **a** Meltwater pooling above frozen ground at the snout of Midtre Lovénbreen glacier, on February 26^th^, 2025. **b** Extensive refreezing of meltwater over the exposed tundra in Ny-Ålesund, on February 28^th^, 2025. **c** A snow pit on Autre Brøggerbreen (78.89410° N, 11.85388° E) on March 1^st^, 2025. The snow depth (from the snow surface to the glacier ice surface) was 60 cm, with multiple ice layers throughout the snowpack. **d** A patch of exposed and awakened tundra during interstitial soil gas sampling, on February 27^th^, 2025. Soil temperature was ~ 1 °C at the soil surface, and the ground was thawed to a depth of up to 5 cm in exposed areas. Photo credits (**a**) James Bradley and Laura Molares Moncayo. **b** James Bradley. **c** Jacopo Pasotti, and (**d**) Donato Giovannelli.

Ny-Ålesund has been a hub for scientific research in the high Arctic for the last five decades^7^. Researchers worldwide come to the remote Arctic outpost to study and monitor terrestrial and marine ecosystems, glaciers, and atmospheric processes, collecting vital information about the functioning of Arctic systems and their responses to ongoing anthropogenic pressures. Winter warming is leading to profound impacts on the Arctic system, and the changes are becoming evident. During this year’s winter field campaign, we directly observed meltwater pooling above frozen ground, forming vast temporary lakes (Fig. 2a). Glacier-fed streams and rivers that usually remain frozen until springtime became active. Snow cover on the tundra was reduced to zero across large areas (Fig. 2b). The reduced snow cover led to greater exposure of the bare ground surface (Fig. 2d). Vegetation emerged through the melting snow and ice, displaying green hues typically associated with spring and summer. Blooms of biological activity were widespread across the thawing tundra (Fig. 2d). Surface soils, which are typically frozen solid during this time of the year, thawed such that they were soft enough to be directly sampled with a spoon, rather than digging snow pits to the soil surface and using drills and pickaxes to extract frozen soil samples (which has been necessary during our normal wintertime sampling operations). The uppermost soils of the permafrost active layer (the active layer is the portion of the soil above permafrost that thaws during summer) were free of snow and thawing in multiple areas, further altering the stability of the terrain. At low elevations, warm temperatures and rainfall on the nearby glaciers diminished snow accumulation (Fig. 2c), and multiple ice layers in the remaining snowpack indicated to us that February’s melting event was not a one-off occurrence this winter.

We anticipate that the impact of increasingly severe and frequent winter warming events will be broadly felt across multiple Arctic systems and societies.

In high-latitude soils, microbial respiration during winter is a key component of the annual carbon budget, contributing significantly to year-round CO_2_ efflux despite subzero temperatures^8^. Winter warming events, especially those involving rain-on-snow or melting, alter the physical characteristics of the snowpack and the underlying soil, reducing thermal insulation and exposing soil microorganisms to increased temperature fluctuations and freeze-thaw cycles. These conditions can shift microbial community composition, influence substrate availability, disrupt microbial dormancy patterns, enhance organic matter decomposition rates, damage soil structure and root systems, and alter gas and water diffusion, which ultimately may lead to elevated emissions of greenhouse gases^11–15^. These changes risk provoking a feedback loop in which warming stimulates further microbial activity and greenhouse gas release, which in turn drives additional Arctic warming.

Prolonged periods of above-zero °C temperatures during winter disrupt seasonal timing across Arctic ecosystems, leading to earlier phenological events^16^. During the winter warming event of February 2025, we witnessed premature plant emergence and thawing of surface soils (Fig. 2b, d). Snow cover over the tundra provides thermal insulation to the ground, buffering the soil against extreme cold and moderating fluctuations in temperatures. Winter warming and rain-on-snow events remove this protective layer, exposing tundra vegetation and soil microorganisms to more variable and far colder temperatures during the remaining winter and spring period^17^.

Meltwater induced by winter warming saturates soil pore spaces with water, which then refreezes into ice. Such icings restrict the infiltration of water produced by snowmelt, leading to large surface meltwater pools over the frozen ground (Fig. 1a). The refreezing of this standing water produces a thick icy crust over the tundra (Fig. 2b). These impermeable crusts impede gas exchange between the soil and the atmosphere, and may alter redox conditions, potentially limiting oxygen availability in the soil and shifting microbial processes towards anaerobic pathways, thus increasing methane production and further disrupting biogeochemical processes and the cycling of climate active gasses. Persistent thick ice crusts over the tundra also significantly reduce the availability of winter forage for reindeer and other herbivores, affecting animal health and survival^18^.

Winter warming also changes the thermal dynamics of the soil as it thaws later in the season. Unlike snow, which is an effective insulator, ice conducts heat more efficiently and has different melting and refreezing properties, altering the timing, rate, and depth of soil thaw in spring^19^. This influences the stability of the active layer of permafrost and disrupts hydrological and biogeochemical connectivity between the soil and adjacent systems, affecting upland systems, including glaciers and tundra, as well as downstream systems, including changes to the distribution and quality of water in rivers and lakes, and sediment transported to fjords and coastal oceans^20,21^.

Human communities and infrastructure are also increasingly threatened by winter warming events. Rain-on-snow events, mid-winter snowmelt, and the formation of weak layers in the snowpack — all driven by winter warming — contribute to snowpack instability and increase the risk of avalanches in populated and frequently traveled areas of Svalbard. The impacts of a warmer Arctic climate on infrastructure are also becoming more apparent. For example, many buildings in Ny-Ålesund — including those housing the UK Arctic Research Station and the Dirigibile Italia Arctic station, which served as our scientific bases in February 2025 — have recently been re-supported on new foundations due to instabilities caused by thawing permafrost and a deepening active layer. As winter warming events become more common, similar challenges will be faced increasingly frequently and more severely by communities and research operations throughout the Arctic.

Of all the seasons, winter is experiencing the most rapid warming, but it is also the season for which the systems and processes are the least documented and understood. While some year-round datasets are available from specific sites and systems in the Ny-Ålesund area (such as the Bayelva permafrost observatory^22^), there remains a notable sparseness of winter and year-round data for Arctic systems, making projections of the likely impacts of winter warming challenging. Moreover, extreme weather and climate events and their impacts can be shaped and amplified by complex interactions between physical drivers and societal factors, making their impacts difficult to disentangle and forecast^23^. The immediate and longer-term effects of winter warming events are still largely undocumented, and years of observations may be needed to assess the changes that are induced. However, there seems to be no doubt that changes to snow and frozen ground across Arctic landscapes produce cascading effects on permafrost thaw, snow and ice melt, and ecological processes. Although the recent thaw event of February 2025 was not an isolated occurrence, witnessing it in real time served as a reminder of the accelerating pace of change, and made us wonder if we have been too cautious with our climate warnings. Winter warming in the Arctic has long reached melting point and is reshaping Arctic landscapes. These winter warming events are seen by many as anomalies, but this is the new Arctic.
